# Safety Culture Through Patient Voice: Qualitative Validation of the Patients' Perceptions of Safety Culture Scale (PaPSC) in Cardiology and Cardiothoracic Surgery

**DOI:** 10.1111/hex.70213

**Published:** 2025-03-05

**Authors:** Clara Monaca, Matthias Weigl, Holger Pfaff, Antje Hammer

**Affiliations:** ^1^ Institute of Medical Sociology, Health Services Research, and Rehabilitation Science (IMVR) Faculty of Human Sciences & Faculty of Medicine and University Hospital Cologne, University of Cologne Cologne Germany; ^2^ Faculty of Human Sciences University of Cologne Cologne Germany; ^3^ Institute for Patient Safety University Hospital Bonn Bonn Germany; ^4^ German Association of Paediatric and Adolescent Care Specialists (Berufsverband der Kinder‐ und Jugendärzt*innen e.V., BVKJ) Cologne Germany; ^5^ Faculty of Medicine and University Hospital Cologne, Institute of Medical Sociology, Health Services Research, and Rehabilitation Science (IMVR), Chair of Medical Sociology University of Cologne Cologne Germany

**Keywords:** interpersonal factors, patient perspective, patient safety, patient‐centred care, qualitative validation, safety culture

## Abstract

**Objective:**

The 11‐item Patients' Perception of Safety Culture Scale (PaPSC) measures patients' perceptions of the safety culture within healthcare organizations. While patients can respond to these items, factors such as limited knowledge, unclear roles and insufficient information may influence their assessments. Despite previous research on the PaPSC, no qualitative validation has been conducted. This study addresses this gap by exploring patients' perspectives on safety culture.

**Methods:**

A qualitative, exploratory approach was adopted, employing problem‐centred interviews with patients from the cardiology and cardiothoracic surgery wards of a tertiary care university hospital. Thematic coding combining deductive and inductive methods was used to identify predefined and emergent themes.

**Results:**

Data saturation was reached after 22 interviews, providing a comprehensive account of patients' experiences. Most patients were able to respond to the PaPSC items, confirming its robustness in assessing safety culture. However, they emphasized interpersonal aspects such as empathy, trust and clear communication, and contextual elements such as the care environment and patient–staff interactions, which are less well represented in standardized instruments. Several items were perceived as ambiguous, highlighting the need for explanatory text to enhance clarity and response accuracy.

**Conclusions:**

This study underscores the value of integrating qualitative methods with standardized tools such as the PaPSC to reveal nuanced aspects of safety culture. Active patient involvement in tool development can improve the comprehensiveness and effectiveness of safety interventions. Ensuring that safety culture assessments accurately reflect patients' experiences and needs contributes to more patient‐centred healthcare practices.

**Patient Contribution:**

Problem‐centred interviews and the Think‐Aloud method were used in this study to ensure active patient participation. Patients contributes by identifying areas where survey items needed further clarification or contextualization, thereby enhancing the validity and usability of the Patients' Perception of Safety Culture Scale (PaPSC). Their feedback also led to the refinements in the study design and tools, underscoring the importance of patient‐centred approaches in healthcare safety research. Although patients were not directly involved in the study design, since the PaPSC scale items were predefined and derived from a prior critical review of existing instruments, their role in this validation study was crucial. The study aimed not to develop new items but to assess the applicability and clarity of an established instrument. The study empowered the patients to share their views openly in a supportive and respectful environment, offering valuable insights for improving safety culture assessment.

## 1 Introduction

Patient involvement is a key but underutilized strategy to improving patient inpatient care. Depending on their health state, patients can actively contribute to their own safety [1, 2]. They are not merely passive recipients of treatment but also continues observers and active participants in the care process [3]. Their unique perspective, combined with their constant presence, enables them to identify potential safety risks early and prevent potential errors. Health literacy studies have shown that patients can support safety interventions and monitor compliance. Through active participation, they can detect and highlight deviations, such as medication errors or procedural inconsistencies [4, 5]. However, many patients find it difficult to openly express safety‐related concerns or questions to healthcare professionals [6, 7]. This reluctance often stems from uncertainty about the role of the care team or a perceived power imbalance in the relationship with healthcare professionals, with time constraints and the perceived low effectiveness of patient engagement activities being key barriers [8]. As a result, patients may hesitate to voice their observations or concerns when they recognize potential risks. Whether patients speak up about safety concerns or critical situations depends largely on the perceived safety culture within the healthcare setting [9]. A positive safety culture fosters openness, transparency and trust and emphasizes that the safety of everyone, patients and staff, is a priority. Developing such a culture is therefore essential for improving patient safety. The importance of including the patient perspective in safety culture assessments is also emphasized in international patient safety initiatives. The WHO Global Action Plan for Patient Safety 2021‐2030 underscores the role of patients and families as active partners in enhancing safety (strategic goal 4) [10].

Safety culture is a subset of the broader organizational culture and encompasses shared values, norms and beliefs that prioritize patient safety as a core objective [11]. Key dimensions include communication, leadership, a blame‐free environment, safety systems, teamwork and workload management [12]. However, these dimensions have predominantly been defined from the perspective of healthcare professionals, while the patient perspective in safety culture assessment has largely been overlooked.

The Patients' Perceptions of Safety Culture Scale (PaPSC) was the first instrument specifically designed to systematically capture the patient perspective on safety culture [13]. In the initial study, this 11‐item scale was administered in an online survey of more than 112,000 individuals with health insurance. Results indicated that respondents were generally able to answer the questions. However, due to limited information and unclear roles, patients had a different perspective on safety measures than healthcare professionals. Despite the high participation rates and good measurement properties of the scale, some critical questions remained unanswered [14]. For example, it was unclear why patients could not rate safety measures in certain areas, leading to frequent ‘not applicable’ responses. One hypothesis is that this issue stems from the design of standardized instruments like the PaPSC, which prioritize simplicity and efficiency to facilitate swift and practical implementation. These characteristics make them particularly valuable in healthcare, as they facilitate comparability of results and seamless integration into different care contexts. However, standardization also presents challenges:

Healthcare is inherently diverse, shaped by cultural, demographic and social factors, as well as variations in care settings [15]. This diversity introduces many nuances that influence the patient experience but may not be fully captured by standardized items. In addition, external factors, such as interactions with healthcare staff, the care environment and patients' individual expectations, play a significant role in shaping their perceptions of safety and their interpretation of specific items. The condensed wording of such items risks overlooking or inadequately representing critical aspects of the patient‐centred perspective.

This study aims to address these research gaps by qualitatively exploring patients' experiences of safety culture in healthcare [16]. Also, the study explores the associations that patients make with specific items and examines which subjective experiences and perceptions are included or excluded. Furthermore, it assesses whether patients interpret the survey questions as intended or whether discrepancies exist between the original purpose of the scales and patients' actual perceptions.

## 2 Methods

This study was conducted in accordance with the Standards for Reporting Qualitative Research (SRQR) guidelines [17] and the Consolidated Criteria for Reporting Qualitative Research (COREQ) checklist [18]. The application of qualitative research methods enabled a comprehensive exploration of patients' needs, perspectives and concerns.

### 2.1 Study Design

This study employed a patient‐centred approach to validate the PaPSC using an exploratory, descriptive design with qualitative methods. To assess content validity, we used problem‐centred interviews that incorporated episodic techniques and concrete examples to illustrate individual patient experiences. This method follows a deductive–inductive approach, allowing for structured yet flexible data collection and analysis [19]. The semi‐structured format enabled participants to freely discuss safety culture issues while maintaining focus [20].

To gain deeper insight into patients' cognitive processes, we also used the Think‐Aloud method, which required participants to verbalize their thoughts while answering survey items. This approach helped identify misunderstandings, ambiguities and contextual associations. The Think‐Aloud approach complemented the semi‐structured interviews and provided real‐time data on how patients interpreted and processed survey questions [21, 22].

A semi‐structured interview guide ensured a coherent flow of information, based on the PaPSC items. Participants had the opportunity to ask questions and share positive and negative experiences. A combined deductive‐inductive approach structured the interviews while allowing unique insights to emerge [23]. All interviews were conducted by the lead author (C.M.).

After each interview, a postscript documented contextual factors, subjective insights and environmental influences [24]. Participants could withdraw from the study at any time without providing a reason. To enhance internal validation, problem‐centred interviews ensured that the PaPSC items were both theoretically sound and practically understandable (Online Resource 1: Interview Guide). All interviews were audio‐recorded and transcribed in full. Sociodemographic data were collected using the *Demographic Standards for Personal Oral and Written Survey* forms. Direct questions (e.g., nationality) were asked, while for other variables (e.g., level of education, employment status), participants selected the most appropriate response from a predefined list of options [25].

### 2.2 Data Collection

Participants were recruited over a 3‐month period (June to August 2021) from the general cardiology or cardiothoracic surgery wards of a maximum‐care university hospital in western North Rhine‐Westphalia, Germany. These wards provided optimal research setting due to the variety and complexity of procedures, potential risks of interventions and patients' in‐depth knowledge of hospital processes. A purposive sampling strategy was employed to maximize diversity in age, gender, diagnoses and treatment modalities (Table 1). Patients were excluded if they did not meet the inclusion criteria, did not provide consent, were critically ill, or had cognitive impairments.

**Table 1:** Inclusion criteria.

Characteristic	Criteria
Age	Over 18 years
Language skills	Very good German language skills
Department	Cardiology and Cardiothoracic Surgery
Time of questioning	Cardiothoracic Surgery: From the 2nd postoperative day
	Cardiology: 2nd day after admission

To ensure data saturation, we aimed to recruit as many participants as necessary to capture a wide range of patient perspectives. Data saturation was defined as the point at which no new themes, concepts or variations emerged from additional interviews, indicating that further data collection would no longer yield meaningful new insights. At this stage, data collection was concluded.

### 2.3 Procedure and Sample Information

Initial contact with potential interview participants was made during their inpatient stay on the general cardiology and cardiothoracic surgery wards. Following the morning ward round, nursing staff identified eligible patients and provided them with an information leaflet about the study and informed the lead author. The lead author then provided further details, explaining voluntary participation, data protection and study procedures. Patients were given sufficient time to consider their participation and discuss with the decision with their relatives. Interviews were conducted either in the patient's room or in a private area of the ward, depending on the patient's mobility and preference. Before each interview, the study aims and procedures were reiterated, and participants provided both written and audio‐recorded consent.

### 2.4 Ethical Considerations

The ethical implications of the study were carefully considered during the planning and execution phases. The study was conducted in accordance with the principles of the Declaration of Helsinki and the European Data Protection Law. The process evaluation was approved by the Ethics Committee of the Medical Faculty of the University of Cologne (No. 20‐1490_1 on December 21, 2020). The study is registered in the German Clinical Trials Register (DRKS) under the DRKS ID: DRKS00022778. The DRKS has been designated as a WHO primary registry since October 2008. Data processing followed the legal principles set out in the General Data Protection Regulation [26].

Following Mayer's [27] guidelines, interviews were conducted in a comfortable, undisturbed environment to ensure a respectful and open dialogue. Effective interview techniques included demonstrating genuine interest, avoiding multiple questions at once, and encouraging participants to share their thoughts freely. No invasive measures were used during the interviews; however, sensitive topics were discussed. To support participants, all patients were offered access to a psychologically trained counselling team. Discussing adverse events can provide relief, foster a sense of being heard, and contribute to a safer hospital environment. Patients may also find the interviews redemptive, viewing them as an opportunity to voice concerns and initiate change that will ultimately benefit future patients.

In addition, the following ethical considerations were addressed:

- Voluntary participation: Patients were reminded that participation was voluntary and could be discontinued at any time without consequence.
- Confidentiality: If patients reported adverse events, they were encouraged to do so confidentially through the hospital's compliments and complaints system, with relevant contact information provided. Absolute confidentiality was assured by the interviewer.
- Protection of hospital staff: Measures were taken to ensure that ward teams were not sanctioned based on the study findings.
- COVID‐19 precautions: All relevant safety protocols were strictly followed.

### 2.5 Analysis

The pseudonymized transcripts were analysed using MAXQDA Analytics Pro 2024 [28], a robust tool for transcribing and analysing qualitative data.

Following the methodology of Kuckartz and Rädiker [29], a deductive‐inductive content analysis was conducted. Initially, the principal investigator immersed himself in the data, creating memos, summarizing case studies and integrating field notes. A preliminary deductive‐inductive category system was developed based on themes from the interview guide, PaPSC items, and an initial text analysis. All transcripts were analysed according to the interview questions outlined in the interview guide. For the PaPSC, individual questions were examined in detail, and subdimensions or categories assigned to each item to better capture the content‐related facets of the respective items. If shifts in thinking occurred during the interviews, the corresponding items were subsequently reassigned to ensure a coherent categorization of content. The coding process was overseen by C.M., with A.H. providing valuable input on refining the category system and interpreting interview excerpts. To ensure objectivity, we also sought feedback from an expert panel. Patient quotations, translated from German to English, are presented alongside pseudonyms to underscore the findings of this study.

### 2.6 Reflexivity and Reliability

To ensure study quality, careful attention was given to credibility, transferability, reliability and confirmability. A comprehensive account of the study's objectives, context, recruitment and data analysis was provided [30]. All interviews were conducted and transcribed by the same author (C.M.), which enhanced reliability, particularly in segments with audio challenges. The interdisciplinary approach enabled a rigorous examination of the findings and generated new insights. This collaboration strengthened the validity and reliability of the results. Following multiple rounds of discussion, the authors collectively determined the most reliable interpretation of the data while also considering alternative perspectives.

## 3 Results

The results begin with descriptive analyses, providing an overview of participants' demographics and key characteristics to contextualize the findings.

The study sample comprised 22 interviews, including 10 conducted in the cardiology ward and 12 in the cardiothoracic surgery ward. Data saturation was achieved after the 22 interviews, as no new findings emerged [31].

### 3.1 Sample Characterization

The interview duration ranged from 16 to 102 min, with an average length of 47 min. The longest transcript contained 11.526 words (Table 2).

**Table 2:** Details of the interviewed patients.

Interview	Gender	Age range	Specialty	Words interview
I_01_KAR	Male	60–69	Cardiology	5.077
I_02_KAR	Male	60–69	Cardiology	5.681
I_03_KCH	Male	40–49	Cardiothoracic surgery	5.531
I_04_KCH	Male	50–59	Cardiothoracic surgery	4.394
I_05_KCH	Male	50–59	Cardiothoracic surgery	11.384
I_06_KAR	Female	70–79	Cardiology	2.069
I_07_KAR	Female	70–79	Cardiology	3.800
I_08_KAR	Female	60–69	Cardiology	1.796
I_09_KCH	Female	60–69	Cardiothoracic surgery	3.862
I_10_KAR	Male	70–79	Cardiology	1.999
I_11_KCH	Male	30–40	Cardiothoracic surgery	3.429
I_12_KCH	Male	50–59	Cardiothoracic surgery	2.875
I_13_KCH	Male	50–59	Cardiothoracic surgery	3.199
I_14_KCH	Male	60–69	Cardiothoracic surgery	5.417
I_15_KCH	Female	> 80	Cardiothoracic surgery	2.619
I_16_KCH	Female	60–69	Cardiothoracic surgery	3.870
I_17_KCH	Female	70–79	Cardiothoracic surgery	2.049
I_18_KCH	Female	50–59	Cardiothoracic surgery	5.641
I_19_KAR	Male	60–69	Cardiology	11.526
I_20_KAR	Female	60–69	Cardiology	1.689
I_21_KAR	Male	70–79	Cardiology	8.335
I_22_KAR	Male	50–59	Cardiology	4.225

Table 3 provides an overview of the sociodemographic characteristics of the 22 participants. The gender distribution shows that 13 participants (59%) were male, while 9 (41%) were female. The mean age of respondents fell predominantly between 50 and 79 years, with the 60–69 age group exhibiting the highest representation (*n* = 8; 36%). Nearly all participants (*n* = 20; 91%) were born in Germany and hold German citizenship. The majority of respondents were married (*n* = 10; 46%) and resided in a two‐person household (*n* = 12; 55%). In terms of professional qualifications, 13 (59%) completed vocational training, and the majority were already retired (*n* = 11; 50%). Regarding monthly household income, the majority of respondents reported earnings between €1.000 and €4.000.

**Table 3:** Samples.

Characteristic	n (%)
Total number	22 (100)
*Gender*	
Male	13 (59)
Female	9 (41)
Diverse	—
*Age groups*	
30–39	1 (5)
40–49	1 (5)
50–59	6 (27)
60–69	8 (36)
70–79	5 (23)
> 80	1 (5)
*Country of birth*	
Germany	20 (91)
Asia	2 (9)
*Nationality*	
German	22 (100)
Additional nationality	1 (5)
*Country of birth of the father/mother*	
Germany	19 (86)
Asia	2 (9)
Europe	1 (5)
*Marital status*	
Married	10 (46)
Single	5 (23)
Divorced	4 (18)
Widowed	3 (14)
*Educational attainment*	
Secondary school	5 (23)
Middle school	8 (36)
Higher education entrance qualification	1 (5)
High school diploma	8 (36)
*Professional qualifications*	
No professional qualification	1 (5)
Completed vocational training	13 (59)
+ additional qualifications	6 (27)
One‐ to three‐year training	4 (18)
Diploma, Master, Magister, State examination	4 (18)
*Employment status*	
Employed (full‐time or part‐time)	8 (36)
Self‐employed or freelancers	2 (9)
Retired/pensioner	11 (50)
Unable to work	2 (9)
*Household size*	
Single‐person household	8 (36)
Two‐person household	12 (55)
Three‐person household	—
Four‐person household	1 (5)
Five‐person household	1 (5)
*Monthly household income*	
< 1.000 €	1 (5)
1.000 € to < 2.000 €	5 (23)
2.000 € to < 3.000 €	2 (9)
3.000 € to < 4.000 €	6 (27)
4.000 € to < 5.000 €	—
5.000 € to < 7.500 €	1 (5)
7.500 € to < 10.000 €	—
> 10.000 €	2 (9)
No information	5 (23)

### 3.2 Safety Culture Coding Patterns and Key Patient Priorities

The coding frequency of the interviews suggests that certain items were particularly well understood and represented key patient concerns. For example, the item ‘During the whole hospital stay, I felt I was in “safe hands.”’ was coded 242 times. Similarly, the item ‘I had the impression that patient safety was always a top priority’. was frequently mentioned, with 142 codes. In addition, 112 codes for ‘I always knew who was responsible for my treatment and care’ indicate that patients valued clarity regarding accountability in their care. In contrast, fewer codes were found for items such as ‘Staff freely spoke up whenever they had the impression that something was amiss’. (58 codes), ‘The information exchange between physicians and nurses. was very smooth’. (74 codes), and ‘The different services (ward, X‐ray, physiotherapy, etc.) are well coordinated’ (79 codes).

Table 4 presents the assigned codes and subcodes for each PaPSC item. In addition, it includes two patient quotations and a key message. The frequency of coding for each specific PaPSC item is also displayed.

**Table 4:** Overview of items, categories and key messages.

		Example 1	Example 2		Key message
Item	**During the whole hospital stay, I felt I was in ‘safe hands’.**				*Amount of coding's: 242*
Code	Satisfying Individual Needs				
		‘… that I am treated quite well by the pain. My pain level is bearable. And I have the feeling that the painkillers are definitely well adjusted’. (I_11_KCH)	‘It was very painful for me. And only a doctor was allowed to take it out’. (I_11_KCH)		Patients feel safe when their pain is managed, and their medication is adjusted to ensure comfort.
Code	Organizational Aspects and Processes ‐ Waiting Time				
		‘If it takes too long at some point, you obviously have to follow up. I think the important thing is knowing that if you have any problem, you can ring the bell. Otherwise, you really can't help yourself’. (I_14_KCH)	‘Unfortunately, it took two and a half hours to have a doctor come. I was like, “Hey, that's a ten‐second mission”. It would have been cool if it had been a little faster’. (I_11_KCH)		Prompt responses to call bells and requests increase patients' sense of safety.
Code	Safety and Assistance				
		‘And it always felt like everything was going well, they were doing it right, and I was well cared for’. (I_02_KAR)	‘On the one hand, uh, I felt like I was in the forecourt of hell here’. (I_19_KAR)		Patients need to feel they're in good hands to feel secure.
Subcode	Communicating and Interacting with Employees				
		‘I once asked something about urine or blood, and he took care of it directly. I wasn't just brushed off’. (I_03_KCH)	‘I ask, “What do I have to take this for”, “I don't know”. These are the kinds of uncertainties you sometimes feel’. (I_16_KCH)		Staff attentiveness and helpfulness make patients feel safe and well cared for.
Item	**I had the impression that patient safety was always a top priority.**				*Amount of coding's: 142*
Code	Patient‐centred care				
		‘I can't say, “No, he didn't treat me on short notice because he'd rather have a coffee” or anything like that, so I haven't experienced that here’. (I_11_KCH)	‘And I wasn't explained afterward how the procedure went, why I had a mental blackout, why I was on the ward for so long, and ultimately I don't know why everything happened the way it did’. (I_18_KCH)		Patient‐centred care that addresses individual needs improves patient safety.
Subcode	Professional‐patient Trust				
		‘And that they know exactly how to tackle one and so on. that just gives you the feeling of security. So, that's how it works for me in any case. There's definitely a lot of head and understanding at work, you can tell’. (I_05_KCH)	‘Well, I've also seen when unforeseeable things happen on the ward, things that are not predictable. And then you're no longer as protected as with an organized surgery here’. (I_18_KCH)		Patient trust in healthcare professionals affects their perception of safety.
Subcode	Safety Culture and Transparency				
		‘…and somebody forgot to turn it back on. Of course, that immediately caused stress here. “How can this happen?”’. (I_13_KCH)	‘The whole system is human, of course, and therefore prone to error. And you have to be a bit careful about that’. (I_14_KCH)		Transparent communication is key to building trust in the safety culture.
Subcode	Compassion and Understanding for Patients				
		‘I know that if something comes up, I will get assistance. Therefore, I never feel like I'm being left alone’. (I_12_KCH)	‘They don't have time for it, and that's where I see the big problem. I don't see the problem as being that the people aren't willing’. (I_04_KCH)		Empathy and understanding are key to improving safety.
Code	Staffing and working conditions				
		‘So, when someone is new, it's immediately noticeable. You can tell who the senior is and who the junior is. There's someone who is learning and, hopefully, there's another person who is explaining things to them’. (I_14_KCH)	‘Sometimes there is a sense of lethargy, like just going through the motions of ABC, without addressing what the patient actually needs at that moment. It feels like they are just following a standard procedure and not able or willing to address individual concerns’. (I_22_KAR)		A shortage of staff limits personalized patient care and attention.
Code	Communication and information flow				
		‘Not only, e.g., is blood pressure monitored and so on, but also people are asked how they are feeling, whether they have dizziness, and if they are experiencing any pain. This means that people who might have minor issues are somewhat encouraged to share them’. (I_19_KAR)	‘Later, when I had to inform the nurses about my appointment, which they were unaware of, it became clear that there was an issue with communication. I think it involves communication among the doctors, nurses, and coordination. Something seems to be going wrong there, but I can't determine exactly what the problem is’. (I_22_KAR)		Effective communication between staff and patients increases the sense of safety.
Item	**The information exchange between physicians and nurses was very smooth.**				*Amount of coding's: 74*
Code	Expertise and trust				
		‘They are very good, very competent. The people, they already know what they are doing, they also know, what's coming up’. (I_04_KCH)	‘Of course, there are also differences in quality, just like with nursing staff. Logically’. (I_01_KAR)		Patients' perceptions of provider competence and trust influence their sense of safety.
Code	Communication				
Subcode	Quality of Communication				
		‘It is almost perfect, and that is good. But that only has to do with communication’. (I_01_KAR)	‘And then we maybe have doctors who didn't properly understand what the professor meant, and they pass it on to the nurse, who doesn't speak German anyway, and then you just have chaos. That is also a matter of safety’. (I_01_KAR)		Understandable communication improves patient safety, while language barriers increase safety risks.
Subcode	Patient‐centred communication				
		‘Like this morning, I had one more tablet. “Here”, I asked the nurses, “why do I have to take this?” “You have to take it because of this and that”’. (I_08_KAR)	‘So, I would have expected someone to say, “Mr. (*Last Name*), we need to adjust these tablets, and this absolutely has to be done”. And, and, and yes, I understand that. But then to just be given this without explanation? Pff’. (I_04_KCH)		Clear, proactive communication about treatment decisions increases patient understanding.
Subcode	Team Communication				
		‘But I have the feeling that the nursing staff already knows what problems and complaints I have and that they coordinate with each other**’**. (I_11_KCH)	‘No, well, it's often the case that then information somehow gets forgotten and isn't passed on**’**. (I_05_KCH)		Good team communication ensures a common understanding of patient concerns.
Item	**The physicians were well informed about my history and current medical condition and treatment.**				*Amount of coding's: 92*
Code	Medical record access				
		‘I had brought documents to this conversation from preliminary examinations and other reports, etc., which all ended up in my file’. (I_02_KAR)	‘I had the impression that they first had to figure out, “Who is he anyway?” Well, that was probably because my documents were probably again somewhere else’. (I_12_KCH)		Timely access to medical records is crucial to providing informed and effective patient care.
Subcode	Interinstitutional information exchange				
		‘I came home from (*name of the previous hospital*). I had already received a call from here to schedule an appointment. So, everything went very well, very quickly. The professor transferred everything here with films’. (I_17_KCH)	‘So, I came here as an emergency. But I was already registered here before. And the documents were there, and the doctors were also informed’. (I_06_KAR)		Seamless, timely patient care requires efficient information exchange.
Subcode	Digital Resource Availability and Use				
		‘These things are also in the computer. Anyone can look them up, and most people do’. (I_01_KAR)	‘There are a few ignorant ones who don't check the computer. Then you always have to tell them everything’. (I_01_KAR)		Digital resources improve access to patient information and care coordination.
Code	Patient Perception of Physician Expertise				
		‘They really need to know my history, otherwise they might not be able to answer me. If I say something, the doctor knows why and for what reason, and I feel that they know that’. (I_07_KAR)	‘The same questions are asked over and over again. Of course, they can't always read everything, so they ask because it's easier to ask than to read thoroughly. In essence, this means that they are not necessarily well informed about the medical history’. (I_22_KAR)		Patients' perceptions of competence depend on doctors' understanding of their medical history.
Subcode	Interaction with physicians				
		‘What I said to the people who came here. From my medical history, I noticed that they paid attention to what I said’. (I_09_KCH)	‘I have the problem that sometimes the doctor explains something to me, and afterward, I don't even dare to ask again. For him, things get so mixed up between Latin and German that I then think, “What was that now?” Then he says, “What do you mean?” Then he starts all over again in the same way, and I don't understand anything’. (I_21_KAR)		Good doctor‐patient communication means hearing and explaining things clearly.
Item	**The nurses were well informed about my history and current medical condition and treatment.**				*Amount of coding's: 96*
Code	Medical record access				
		‘And then this very nice nurse somehow came and brought me something else. Then she said, ‘I took the liberty of looking at your file, where the bypass needs to be done – that's a very critical spot, it has nothing to do with minimally invasive procedures. You can't risk that’. I had the impression that she was interested in me’. (I_02_KAR)	‘They probably know my medical history. They just need to look at my patient file. But whether they really know it ad hoc when they're standing by my bed, I don't think so’. (I_21_KAR)		Access to medical records is crucial for providing informed and personalized patient care.
Subcode	Digital Resource Availability and Use				
		‘When someone from the nursing staff here asks me, “How are you?” I say, “I'm doing well”. Then they check the machine. Then they say: “But you must have been feeling unwell”. I did have a strange feeling during the night, but now I'm feeling fine again. Then that was probably it’. (I_21_KAR)	‘So, this is the first time that I'm not hooked up to wires. I always had to carry around this thing, I don't know what it's called. In the end, when I was still in the ICU, I was completely wired up, with all the monitors hanging there. So, the monitoring is really excellent’. (I_15_KCH)		Monitoring technology ensures that patient issues are detected early and appropriately addressed.
Code	Patient Perception of Nurse Expertise				
		‘The nurse looks here, “Okay, bypass surgeries”. They have so much experience that they can also see, “How long ago was that approximately? How far along is he already? He's already walking around. He only has the EKG and nothing more”. They have a certain level of experience from which they can deduce a lot’. (I_14_KCH)	‘Actually, I have more the impression that they are just doing their job. And I don't necessarily know if they are fully informed about the entire medical history. I don't know. No, I don't really think so’. (I_13_KCH)		Patients' views of nurses' expertise are shaped by their knowledge of the patient's medical history.
Subcode	Patient‐centred care				
		‘And when someone comes in and has an eye for the patient and how they're lying there. Or just comes in and asks, “How are you? Are you thirsty? Does anything hurt?” That is already worth a lot’. (I_05_KCH)	‘But I would have expected, in some things, that they would talk to me. And yes, maybe there are people with whom you can't discuss things, who simply aren't able to understand things. But I would claim that I am capable, or would be capable, of understanding and grasping things. And then, sometimes, I would have expected them to simply talk to me’. (I_04_KCH)		Personalized attention and clear communication are essential for patient well‐being and understanding.
Subcode	Interaction with nurses				
		‘Because they respond to it and also tell you how things are done. And then they actually always respond to it very nicely. They do sometimes talk about various things, though briefly, of course, within the time they have. But you still get the impression that they are responding to me’. (I_22_KAR)	‘Because there's no discussion about the medical history. No one asks anything. They just act’. (I_20_KAR)		Interaction with nurses is shaped by their attentiveness to the patient's concerns and the clarity of their explanations.
Item	**After handover (shift change, transfer), staff knew all relevant information necessary for my care.**				*Amount of coding's: 81*
Code	Expertise and trust				
		‘They just come in here and take the right actions’. (I_10_KAR)	‘I always said better safe than sorry. I always rehearsed a bit and asked questions, so of course they have a lot of topics. And then I always made sure that they got into it a bit’. (I_14_KCH)		Competent acting and thoughtful questioning foster trust between patients and healthcare providers.
Subcode	Staff continuity				
		‘And it would, of course, be great (…) (*Name of the nurse*), who has now done three‐night shifts. Then you know them, you know how they react, and you know how they do things’. (I_14_KCH)	‘But of course you can't have a permanent contact person, that's clear. You can't guarantee that with shift work’. (I_14_KCH)		Continuity of caregivers fosters trust and confidence in patient care.
Code	Communication and information flow				
		‘I don't have to explain my complaints anew to everyone here. Essentially, they always ask follow‐up questions based on the last known condition’. (I_11_KCH)	‘Things do get missed from time to time (…) and little things are occasionally forgotten, but that's more or less normal. However, I don't have the impression that anything serious has been forgotten so far’. (I_22_KAR)		The flow of information is generally reliable, even if small details are sometimes forgotten.
Code	Patient expectations				
		‘The more important thing is that the person who comes in gets a good overview. And then bring their experience to the patient and see, ask, “anything wrong?” or something like that’. (I_05_KCH)	‘Four times I told a nurse in the morning that I needed an appointment with the dentist, “I'll take care of it”, they said. I don't know how the process works. But I asked three or four times, and nothing happened’. (I_05_KCH)		Patients expect staff to be knowledgeable about their condition and responsive to their needs.
Item	**Physicians and nurses worked together as a well‐rehearsed team.**				*Amount of coding's: 86*
Code	Effective teamwork				
		‘Yes, absolutely. It makes me happy to see that there is almost a friendly relationship between the nursing staff and the doctors. When I see how well they get along and work hand in hand, I really like that’. (I_05_KCH)	‘I believe there is room for improvement here. I suspect it's due to a certain turnover of staff and also the age or age structure. There is a lot of young staff here, and even the ward doctors seem to be very young and make a youthful impression, which also has a different influence on the teamwork with the nursing staff’. (I_19_KAR)		Well‐managed age structures and harmonious employees foster effective teamwork.
Code	Communication				
		‘It starts with the way they address each other and the fact that they use informal language, on a first‐name basis. I think that is a good sign’. (I_10_KAR)	‘Always with the exception of those I mentioned, like intelligence level and language skills. The staff should first be sent to a language school. For me, it's more of a collaboration rather than teamwork’. (I_01_KAR)		Good communication, language skills, and respect promote teamwork.
Code	Hierarchy and role distribution				
		‘Let's put it this way: with the senior physician, you can definitely notice the difference between them and the nursing staff. In the case of the ward doctor and the nursing staff, you rarely notice the difference, and even less often’. (I_21_KAR)	‘And I hope that one day it will truly be a thing of the past for doctors to think they are somehow better just because they studied. And that the work of nurses and caregivers is not as important’. (I_05_KCH)		The notion that physicians are superior because of their education is seen as outdated.
Item	**The different services (ward, X‐ray, physiotherapy, etc.) are well coordinated.**				*Amount of coding's: 79*
Code	Process Flow				
		‘It starts here on the ward with the escort service, which drives you back and forth. And it works well. You arrive, are greeted, and then taken back out, and most of the time, the driver is already there again. Even though the distances are quite long, all the way to the X‐ray, I must say, for the distance, it's pretty well‐timed. I have no idea how they manage that’. (I_11_KCH)	‘This Heart Center is a good example because they have certain parts of the organization under their own control, like a defibrillator, some tests downstairs, echocardiograms. That's organized independently. But as soon as that organization is dependent on others in that building, like X‐ray, for example, over in the main hospital building, then the priorities are set according to what they want or needs. You're dependent on them. There's no collaboration at all’. (I_01_KAR)		Smooth patient flow contributes to efficient processes but is dependent on external departments.
Subcode	Waiting time				
		‘I was driven to the X‐ray in the main hospital building. Then they pushed me to the side. “Okay, you'll need to prepare for a longer stay here”. The lady called me in, and after 2 min, I was done. That's the range, of course’. (I_14_KCH)	‘Yesterday I had to go for a sonogram. They picked me up just before three and I was back here just before six. I spent almost 3 h lying in the hallway. It wasn't great, of course. Maybe they could schedule things a little better. When I was wheeled over there were six beds in the hallway. But well, I didn't have anything else planned for the day anyway (*laughs*). (…) The examination itself took maybe 15 min, and then I spent at least another 20 min in the hallway until someone came to get me. But okay, it was a bit of a mess’. (I_13_KCH)		Long waits highlight the need for better scheduling.
Code	Coordination between functional areas				
		‘In terms of logistics, it works very well, I really have to say. I never thought, “Oh my God, you still haven't been to the X‐ray”, or “Where's the physiotherapist?” No, everything has gone smoothly. From a logistical point of view, everything has been going well for me here’. (I_18_KCH)	‘I was taken to sonography, got checked in, but I wasn't registered. Why? They took me to regular sonography, but I was supposed to go to emergency sonography. So, I was driven to emergency sonography, which means you're just sitting there, feeling out of place (…) When I was done, the driver finally came, but he had been looking for me in the regular sonography, and now an emergency sonography had been done. So, the whole thing took more than an hour instead of 20 min’. (I_19_KAR)		Efficient coordination ensures smooth logistics, but miscommunication causes delays and disruption.
Item	**I always knew who was responsible for my treatment and care.**				*Amount of coding's: 112*
Code	System confidence				
		‘Because it's like a red thread running through the entire time you're here. One thing leads to another, and it's always the same. And that's how you realize how it works’. (I_09_KCH)	‘But once I'm inside the hospital, I really don't care who comes in and treats me, because I just trust that they know what they're doing’. (I_05_KCH)		Consistent processes and confidence in the system foster patient satisfaction with the quality of care.
Code	Clarity of responsibilities				
		‘Yes, the first team had already introduced themselves and talked with me. That was great. I had also been in the ICU for a few hours before, and I found that very well done’. (I_14_KCH)	‘Hang up boards with pictures, then I can say, “She said this”, or “He or she always does it like that.” But as it is, it's just impossible. I'm not great at memorizing or remembering names anyway. But if I'm also under medication here, then it's a lost cause’. (I_05_KCH)		Clear staff roles improve patient experience and hinder confusion.
Subcode	Physicians				
		‘I had a doctor here, unfortunately I don't remember her name, a young doctor, and she was responsible for me the whole time. Occasionally, she consulted with another doctor, but she was essentially our main contact person. She always had time for all our questions’. (I_12_KCH)	‘As a patient, you don't know who's doing the rounds today. How am I supposed to know? Sometimes they're in the ICU, then they're here, then they have a day off, then they're on night duty, then they're on vacation, or suddenly they're on another ward. No, you never know’. (I_18_KCH)		Consistent physician contact improves patient communication and trust.
Subcode	Nursing Staff				
		‘But many do say, “See you tomorrow, I have the early shift”. They don't have to say that, but it's nice. When you know, “So‐and‐so is here tonight”, you feel a bit better, knowing who's looking after you. I have to say, when I know, e.g., that (*name of staff member*) is on night duty, then I think, “All right, she has everything under control”. And that has always been reassuring to me’. (I_18_KCH)	‘Honestly, I don't really know which nurse is giving me injections or who can just change a plaster. I have to say, it's a bit hard to figure out’. (I_11_KCH)		Consistency in nursing staff provides comfort and reassurance to patients.
Code	Staff Changes/Shift Work				
		‘Yes, it keeps changing. I think it changes because they are understaffed here. Okay, you might build a relationship with someone, everyone is great. But I've been here for two and a half weeks, and there are only three or four people who regularly check my file and come talk to me. Unfortunately, here, a lot of people rotate, and I think it's because they're understaffed’.(I_03_KCH)	‘When there are people, you've never seen before, who suddenly come to take blood at night, you're like, ‘Who is this again now?’ That makes you feel a bit uneasy’. (I_18_KCH)		Frequent staff turnover due to understaffing disrupts patient‐staff relations and safety.
Item	**Staff freely spoke up whenever they had the impression that something was amiss.**				*Amount of coding's: 58*
Code	Response to problems				
		‘This morning the nurse came again, and I looked after her as she left. “(*Last name*), is everything alright?” “Why? Do I look like something's wrong?” “No, no, no, I just thought maybe you had a question or something”’. (I_12_KCH)	‘This morning, the staff also said, “Of course, it's frustrating for you, (*Last name*). I'll follow up on that. It can't be that you've been waiting for two and a half hours and are in so much pain”. That really gives you the feeling that the nursing staff is dedicated to individual cases’. (I_11_KCH)		Staff who respond to patient concerns and solve problems proactively build trust and reassure patients.
Code	Informal communication between staff				
		‘So, if they have to sort something out, they do it among themselves anyway. You might overhear them on the hallway once in a while, trying to clarify something, but always in a very civil tone. You wouldn't really notice that two people are having a disagreement or anything like that. And that would be saying too much anyway. So, everything runs smoothly’. (I_05_KCH)	‘No, I didn't notice that. But to be honest, I can't recall a situation where several doctors were in the room with me at the same time. So, as for communication between the doctors and the nursing staff or other doctors, I can't really say much about it’. (I_11_KCH)		Informal communication between staff is usually discreet, polite, and not in front of the patient.
Code	Openness in communication				
		‘The examination did not take place today due to the lack of information. This was already known before I was up here. That's why the communication worked, and the doctor addressed it directly, saying it was his mistake and apologizing for it. Unfortunately, we couldn't do it again until tomorrow. That's what he said. I can handle it better when someone is straightforward rather than beating around the bush and potentially leaving everything unresolved’. (I_22_KAR)	‘I'm not involved in that, so I don't know if anything is actually being addressed. I can't judge that from here because I haven't seen any reactions to it’. (I_22_KAR)		Openness and direct communication from staff about issues builds patient trust and helps manage expectations.
Item	**There was always enough qualified staff available.**			*Amount of coding's: 86*	
Code	Availability of qualified staff				
		‘But for me, I had the impression that, yes, there was enough staff and also enough qualified staff. And because I'm in a large hospital, I know that sometimes there are interns there to observe. That's something I take for granted’. (I_12_KCH)	‘When, for example, at night an experienced nurse is working a shift with a completely inexperienced trainee, and maybe it's a particularly tough night for some reason—who knows, maybe because the moon is shining a bit brighter—it's sometimes crazy what happens during those nights. I don't know how they manage it’. (I_05_KCH)		Qualified staff are reassuring, but inexperienced trainees in difficult situations are a cause for concern.
Code	Perception of qualification				
		‘Yes, I think so. Well, the doctor had to explain to a colleague how exactly to press the button on the external pacemaker—twice here and once there. But that's okay, one of them knows how to do it, and the competence is definitely there. Absolutely’. (I_14_KCH)	‘I'll put it this way. From my observation, I'd say, to put it casually—you have the experienced staff, and then you have a few “zero types”, and they somehow get paired together. Depending on the situation, it can lead to the negative side if there are too many “zeros”, and the others are busy. Then you have a problem’. (I_01_KAR)		Patients see staff as qualified, but concerns arise when inexperienced staff are supervised inadequately.

### 3.3 Summary of Content Validation Process for the PaPSC Items

This section summarizes the coding evaluation in relation to the PaPSC items, integrating central themes and key messages to illustrate patients' perceptions of safety and satisfaction during their hospital stay. The codes and subcodes were systematically organized into thematic categories to represent relevant aspects of safety culture from the patient's perspective at different organizational levels.

#### 3.3.1 During the Whole Hospital Stay, I Felt I Was in ‘Safe Hands’

Many patients reported feeling well cared for during their hospitalization, particularly regarding effective pain management and the prompt responsiveness of staff. A sense of safety was closely linked to confidence in staff competence. However, long waiting times and a lack of transparency in information sharing led to feelings of uncertainty for some individuals. Effective communication and staff helpfulness were identified as key factors influencing patients' well‐being.

#### 3.3.2 I Had the Impression That Patient Safety Was Always a Top Priority

Patients frequently expressed a positive perception of safety during their hospitalization, largely due to the expertise and professional care provided by staff. Prompt responses to concerns and transparent communication in the event of errors further reinforce confidence in the hospital's safety culture. However, some respondents noted that staff shortages and the presence of inexperienced personnel could compromise safety. Additionally, it was emphasized that effective communication and individualized care play a crucial role in fostering a sense of safety among patients.

#### 3.3.3 The Information Exchange Between Physicians and Nurses Was Very Smooth

Patients had varying perceptions of the information exchange between physicians and nursing staff. While some commended the staff's competence and effective communication, which fostered a sense of security, others reported challenges, particularly language barriers and inadequate communication. Nevertheless, many emphasize the effective collaboration within the medical team, which contributed to improved coordination and quality of care.

#### 3.3.4 The Physicians Were Well Informed About My History and Current Medical Condition and Treatment

Patients reported mixed experiences regarding how well doctors were informed about their medical histories. While some felt well cared for, benefiting from an efficient exchange of medical records, others expressed concerns that physicians were not always well informed and frequently repeated the same questions. The use of digital resources was generally perceived as beneficial, though not consistently implemented. Patients emphasized that clear and understandable communication was essential for fostering trust in the quality of their care.

#### 3.3.5 The Nurses Were Well Informed About My History and Current Medical Condition and Treatment

Patients' perceptions of nurses' knowledge regarding their medical history and treatment varied. While some were satisfied with the level of information provided and the nurses' attentiveness, others raised concerns about gaps in communication. Some patients perceive digital resources and technical monitoring as beneficial for tracking their health status. The competence of nursing staff was partially assessed through their interactions and communication with patients. Several respondents expressed a desire for more detailed explanations and conversations about their treatment plans to enhance their perception of care.

#### 3.3.6 After Handover (Shift Change, Transfer), Staff Knew All Relevant Information Necessary for My Care

Following shift changes or handovers, patients typically reported a satisfactory exchange of information and a sense of security in the hands of their care providers. Recurring staff members were perceived as particularly reassuring. However, on rare occasions, minor details were inadvertently overlooked, causing distress, especially when patients' concerns were repeatedly expressed but not addressed.

#### 3.3.7 Physicians and Nurses Worked Together as a Well‐Rehearsed Team

The majority of patients expressed a positive opinion regarding the collaboration between medical practitioners and nursing staff. Many commended the collaborative and amicable teamwork, while some highlighted potential areas for improvement, particularly considering staff transitions and the presence of hierarchical structures. Effective communication and mutual trust were considered essential. However, language barriers and hierarchical differences were perceived as potential obstacles that could impact teamwork effectiveness.

#### 3.3.8 The Different Services (Ward, X‐Ray, Physiotherapy, etc.) Are Well Coordinated

Most patients perceived the coordination between different hospital department, such as the ward, X‐ray department and physiotherapy, is perceived by most patients to be well‐structured and effective. The implementation of organized procedures and smooth interdepartmental collaboration contributed to a positive treatment experience. However, some patients reported delays and confusion, particularly in cases involving external dependencies or insufficient communication between departments. Waiting times, in particular, were identified as a concern, highlighting the need for improvements in scheduling and coordination.

#### 3.3.9 I Always Knew Who Was Responsible for My Treatment and Care

A number of patients reported a high level of confidence in the hospital and its processes, which they felt contributed to their well‐being. The consistency of procedures and clear organizational structure reinforced this trust, as patients perceived that everything was functioning effectively. Clarity regarding responsibilities and effective communication with staff were identified as particularly important factors. Patients expressed gratitude for the transparent delegation of responsibility within the healthcare team, commending the unwavering dedication of medical professionals. However, some patients experienced uncertainty, particularly due to frequent staff changes and unclear responsibilities, which led to confusion.

#### 3.3.10 Staff Freely Spoke up Whenever They Had the Impression That Something Was Amiss

Patients reported varied experiences regarding the openness of staff in addressing problems. Some respondents commended the proactive approach taken by nursing staff, which reinforced their trust in the care provided. An open and transparent communication style was perceived as particularly positive, especially when mistakes were acknowledged with sincerity, Fostering a sense of security. However, some patients expressed frustration over a perceived lack of transparency and clarity in communication, which they felt contributed to uncertainty. In general, fostering open communication increased trust in the care and treatment provided.

#### 3.3.11 There Was Always Enough Qualified Staff Available

Overall, most patients expressed positive sentiments regarding the quality of care they received. However, some questioned staff qualifications, particularly when inexperienced personnel lacked adequate guidance or when existing staff were overworked. While some patients expressed confidence in staff competence and felt well cared for. Others were concerned about the deployment of inexperienced personnel in challenging situations without sufficient support. Although the overall feedback was predominantly positive; the balance between experienced an inexperienced staff was occasionally criticized.

### 3.4 Thematic Summary of Codes and Subcodes

**Figure 1:**
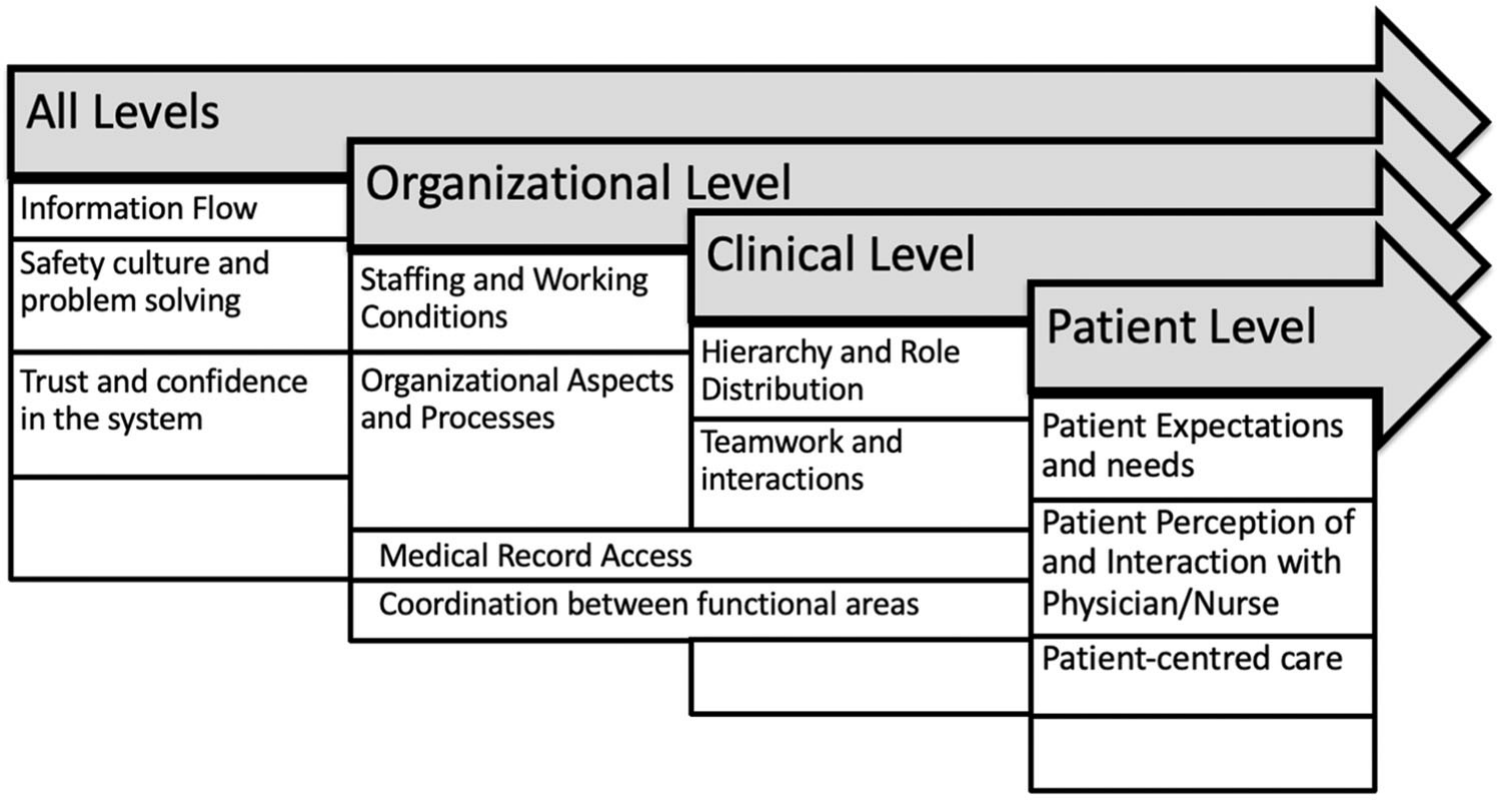
Overview of levels and categories based on thematic coding.

This section builds on the initial identification of codes and subcodes to present a systematic summary and thematic organization. The aim of this approach is to identify the key factors shaping safety culture from the patient's perspective across different organizational levels. Aligning the codes within a structured framework allows for a holistic analysis of both patient‐centred and organizational aspects. Figure 1 provides a visual representation of the categories developed at the All Levels, the Organizational Level, the Clinical Level and the Patient Level.

## 4 Discussion

The PaPSC is an innovative approach that systematically integrates the patient perspective into safety culture assessment. Standardized instruments like the PaPSC are valuable for measuring and comparing safety culture across care settings [32, 33, 34], However, they often fail to fully capture the complexity of patient experiences in healthcare [35]. The healthcare system is inherently characterized by numerous individual nuances that cannot be fully addressed by standardized survey items [36]. Additionally, applying these instruments to diverse patient groups and varied care settings poses a significant challenge [16]. To enhance the quality and validity of such instruments, a thorough understanding of the patient's perspective is essential [37]. Combining standardized scales with qualitative methods can reveal the ‘invisible dimensions’ behind survey items, thereby refining measurement tools and promoting patient‐centred improvements in healthcare [38].

The evaluation and comparison of the quantitative and qualitative results identified several research gaps, including why certain aspects of patient safety culture are difficult to assess [38]. The findings indicate that each PaPSC item represents underlying subdimensions of safety culture. Consequently, additional explanatory text was developed to explicitly include these subdimensions, thereby improving the comprehensibility and applicability of the scale across different patient groups (see Online Resource 2 for the additional explanatory text). For example, the item ‘During the whole hospital stay, I felt I was in “safe hands”’. was coded 242 times, suggesting its importance to many patients. The item with the highest level of agreement in the pilot study was 84,9%. The high coding frequency and strong agreement rate suggest that patients consistently felt reassured and confident in the care they received, emphasizing the importance of trust and safety during their hospital stay. In contrast, the item with the fewest codes was ‘Staff freely spoke up whenever they had the impression that something was amiss’ (58 codings) and also received the highest percentage of ‘not applicable’ responses (25.7%) in the pilot study [13]. This finding suggests that many patients found it difficult to evaluate or comment on this aspect of their care experience.

Another key finding is the identification of ‘semantic fields’ underlying specific survey items, reflecting how patients interpret the same item differently based on their experiences, expectations and context [39]. For example, while one patient may associate ‘safe hands’ as emotional support and empathy, another may interpret it with procedural accuracy or technical competence [40]. These findings underscore the need to account for such variation to refine and improve survey instruments.

The identification of ‘semantic fields’ also highlights the sender‐receiver dynamic between patients and healthcare professionals [41]. Patients respond based on their subjective experiences, whereas healthcare professionals interpret these responses through a clinical lens. These differing perspectives can result in misinterpretations, potentially leading to inaccurate conclusions or missed opportunities for improvement.

To bridge this gap, explanatory materials and training are essential to enhance healthcare professionals' understanding of the patient perspective. Such materials can serve as interpretive guides, ensuring that patient responses are contextualized and effectively inform quality improvement efforts.

Several strengths emerge from this study. First, it is among the first to systematically integrate the patient perspective into safety culture assessment using a validated tool. By combining standardized and qualitative approaches, we explored not only how patients respond to safety culture items, but also why they interpret them in certain ways [42, 43]. This methodological triangulation enhances the validity and applicability of the PaPSC. Second, problem‐centred interviews and the Think‐Aloud method actively involved patients in refining the comprehensibility and contextual relevance of the scale. Potential misinterpretations were minimized and usability across different patient groups was improved. Finally, our findings underscore the importance of patient‐centred safety culture assessments that consider both ‐ technical and relational dimensions of safety [44]. Patients tend to prioritize interpersonal aspects and trust in healthcare professionals over procedural factors [45]. Consistent with previous research trust in healthcare professionals has been shown to have a significant impact on perceived safety and patient satisfaction [46, 47]. The identified codes and sub‐codes are closely in line with findings from other safety culture and patient safety studies [48, 49]. However, it is important to acknowledge that some studies have also identified negative aspects of active patient involvement, such as patients feeling overwhelmed [50]. These findings emphasize the need to view patient involvement not merely as an ideal but as a complex process that includes both positive and potentially burdensome aspects [51].

The PaPSC is a valuable tool for the systematic assessment of patient safety culture from the patient's perspective. Our findings suggest that the applicability and validity of the instrument in different healthcare settings could be further enhanced through targeted refinements. By incorporating qualitative insights into standardized assessments, future studies can improve the accuracy and interpretability of safety culture measures [52].

Integrating the patient perspective into standardized instruments remains a key challenge, particularly when considering diverse patient groups and care contexts. Future safety culture research should further examine how different patient demographics influence perceptions of safety and whether tailored wording of survey questions could reduce variability in responses [53]. Another important focus should be on minimizing communication barriers between patients and healthcare professionals. This includes training programmes to raise staff awareness of the importance of patient involvement and the development of strategies that encourage patients to openly share their observations and concerns [54].

## 5 Limitations

This study has several limitations regarding its scope and generalizability. First, the sample was restricted to cardiology and cardiothoracic surgery patients from a single hospital. Institutional factors may have influenced the findings, limiting their applicability to other hospitals or healthcare settings. Additionally, as the interviews were conducted within a fixed study period, the results reflect a specific point in time, without capturing longitudinal changes in perceptions of safety culture. Moreover, the sample may not fully represent diverse patient groups in terms of age, cultural background or chronic conditions.

Another limitation is that patients were not directly involved in the study design, as the primary aim was to validate an existing instrument rather than develop a new one. The decision to use a predefined scale was based on previous research, in which relevant survey items had already been identified. However, involving patients earlier in the development phase could have further enhanced the instrument's applicability to patients' real‐life experiences.

Qualitative research has inherent limitations, including the potential for interpretation bias, particularly since all interviews were conducted by a single researcher. Despite measures to increase reliability, subjectivity cannot be completely eliminated. While data saturation is a widely accepted concept, it remains somewhat subjective and depends on the diversity of the sample, the research objectives and the depth of analysis. Although it is defined as the point at which no new themes emerge, it does not guarantee that all perspectives have been fully captured. In studies with diverse patient experiences, some nuances may persist. We closely monitored emerging themes and ensured a diverse sample, but limiting the study to two departments may have influenced the point at which saturation was reached. Additionally, as the study focused primarily on the PaPSC, other relevant aspects of patient safety culture may have been overlooked.

## 6 Conclusion

This study advances our understanding of patients' perceptions of safety culture by identifying key dimensions that influence how patients evaluate safety in healthcare settings. By integrating qualitative methods with a standardized measurement tool (PaPSC), we explored not only how patients respond to safety culture items, but also why they interpret them in particular ways. The findings highlight the importance of ensuring that such tools accurately capture the diversity and complexity of patient experiences, rather than reducing them to numerical scores. A key finding is that interpersonal aspects—such as trust, communication and empathy—are central to perceptions of patient safety. While standardized instruments provide valuable benchmarking opportunities, they should be continuously refined to reflect both procedural safety measures and the relational dynamics that affect patient well‐being.

Moreover, this study supports global patient safety initiatives, particularly the WHO Global Action Plan for Patient Safety 2021–2030 (Strategic Goal 4), which emphasizes the active involvement of patients and families in safety improvement efforts [10]. The findings highlight the value of qualitative approaches in filling gaps in standardized assessments and ensuring that safety culture measures are more patient‐centred and context‐sensitive. To build on these findings, future research should expand data collection to include more hospitals and specialties to examine how patient safety culture differs between institutions.

In addition, longitudinal studies could provide insights into how perceptions of safety culture evolve over time and in response to healthcare interventions. By incorporating these findings into future research and practice, healthcare systems can improve the validity of safety culture assessments, strengthen patient‐centred safety strategies and promote a more inclusive and transparent safety culture. This study serves as a foundation for advancing holistic, mixed‐methods frameworks that combine qualitative depth with quantitative rigour to improve patient safety outcomes globally.

## Author Contributions

**Clara Monaca:** conceptualization, methodology, software, validation, formal analysis, investigation, resources, data curation, writing ‐ original draft, writing ‐ review and editing, visualization, project administration, funding acquisition. **Matthias Weigl:** methodology, writing ‐ review and editing, supervision. **Holger Pfaff:** methodology, writing ‐ review and editing, supervision. **Antje Hammer:** conceptualization, methodology, validation, formal analysis, investigation, writing ‐ review and editing, supervision.

## Ethics Statement

Approved by the Ethics Committee of the Medical Faculty of the University of Cologne (No. 20‐1490_1 of December 21, 2020). The study was registered in the German Clinical Trials Register (DRKS) under DRKS ID: DRKS00022778.

## Consent

All participants provided their explicit consent for the anonymized publication of their data. They were informed that their responses would be used in academic publications and were assured that no personally identifiable information would be disclosed. All participants provided informed consent before being included in the study. They were informed of the purpose, methods and implications of the research and were assured of their right to withdraw at any time without consequence. Participants' confidentialities and anonymity were maintained throughout the research process.

## Conflicts of Interest

The authors declare no conflicts of interest.

## Supporting information

Supporting information.

Supporting information.

## Data Availability

The datasets generated and analysed in this study are not publicly available to protect the participants' anonymity and confidentiality. In addition, the participants did not give consent for their data to be shared with third parties.
