# Supplementary material for: Safety Culture Through Patient Voice: Qualitative Validation of the Patients' Perceptions of Safety Culture Scale (PaPSC) in Cardiology and Cardiothoracic Surgery
Source: Health Expect. 2025 Mar 5;28(2):e70213. doi: 10.1111/hex.70213 (PMC11882746; doi:10.1111/hex.70213)
Supplement: Supplementary file 1 — Supporting information. [file HEX-28-e70213-s002.pdf]

Online Resource 1

Article title: Safety culture through patient voice: Qualitative validation of the Patients' Perceptions of Safety Culture Scale (PaPSC) in Cardiology and Cardiothoracic Surgery
Journal name: Health Expectations
Authors: Clara Monaca, Matthias Weigl, Holger Pfaff, Antje Hammer
E-mail address of the corresponding author: cmonaca@smail.uni-koeln.de

---

Interview Guide

Introduction to the topic of safety culture from the patient's perspective

I would like to ask you to think about this or another hospital stay...

- Did you feel in "safe hands" during the whole hospital stay?
  - *Can you describe this in more detail? Can you give some examples?*
  - *(IF APPLICABLE: In what way is this question difficult to answer?)*

  → Enquiries for all questions are the same.

- Did you have the impression that patient safety was always a top priority?
- Did you feel that the information exchange between physicians and nurses was very smooth?
- Did you feel that the physicians were well informed about my history and current medical condition and treatment?
- Did you feel that the nurses were well informed about my history and current medical condition and treatment?
- After handover (shift change, transfer), did you feel that the staff knew all relevant information necessary for your care?
- Did you feel that the doctors and nurses worked together as a well-rehearsed team?
- Did you feel that the different services (ward, x-ray, physiotherapy, etc.) were well coordinated?
  - *Transfers to other departments (e.g., emergency room to ward, intensive care unit)*
- Do you always know who was responsible for your treatment and care?
- Did you feel that Staff freely spoke up whenever they had the impression that something was amiss?
- Did you feel that there was always enough qualified staff available?

Consolidation question

- What does patient safety mean to you?
  - *If you had to explain it to someone, what would you say patient safety involves?*

Involving patients in safety measures (role change)

• Were you asked your name before your operation or procedure?<br>○ *Were you asked your name more than once before the procedure?*<br>○ *How did you feel about this?*<br><br>• What would you do if staff called you by the wrong name?<br>○ *How would you feel?*<br>○ *Have you ever experienced this? Can you describe it?*<br>→ Enquiries for all questions are the same.<br><br>• What would you do if your pillbox contained unfamiliar or missing medicines?<br>• What would you do if you had the feeling that your meal was the wrong one?<br>• What would you do if you had the feeling that your treatment was not the right one?<br><br>• Have you ever noticed anything unusual about another patient?<br>○ *Did you report it to the staff?*
Follow-up question
• Considering all aspects of the hospital: How safe do you feel in this hospital?<br>• What do you think we could do to make you feel safer?
Conclusion
• Is there anything you would like to add or mention that wasn't covered in the interview?
Final clarification
• What has been the most important thing that you have said today in the interview?
Sociodemographic data collection
