# Supplementary material for: Safety Culture Through Patient Voice: Qualitative Validation of the Patients' Perceptions of Safety Culture Scale (PaPSC) in Cardiology and Cardiothoracic Surgery
Source: Health Expect. 2025 Mar 5;28(2):e70213. doi: 10.1111/hex.70213 (PMC11882746; doi:10.1111/hex.70213)
Supplement: Supplementary file 2 — Supporting information. [file HEX-28-e70213-s001.pdf]

Online Resource 2

Article title: Safety culture through patient voice: Qualitative validation of the Patients' Perceptions of Safety Culture Scale (PaPSC) in Cardiology and Cardiothoracic Surgery
Journal name: Health Expectations
Authors: Clara Monaca, Matthias Weigl, Holger Pfaff, Antje Hammer
E-mail address of the corresponding author: cmonaca@smail.uni-koeln.de

---

It is recommended that the following information be included in the item description to ensure that patients are adequately informed of the potential implications of this item.

<u>During the whole hospital stay, I felt I was in “safe hands.”</u>
Explanation: Consider if your pain management was handled well, whether the staff responded promptly to your needs, and if you felt confident in their abilities. Reflect on how the staff’s communication and attention to your concerns contributed to your overall sense of security. Additionally, think about whether any delays or unclear information caused you to feel uncertain or less secure.

<u>I had the impression that patient safety was always a top priority.</u>
Explanation: Consider if the staff consistently demonstrated competence, communicated clearly about any issues, and responded quickly to concerns. Reflect on whether the medical team addressed mistakes openly, provided explanations for treatments, and ensured that your needs were met in a timely manner. Also, consider if any issues like staff shortages or the presence of less experienced staff impacted your sense of safety during your care.

<u>The information exchange between physicians and nurses was very smooth.</u>
Explanation: Consider whether the communication was clear, consistent, and professional. Think about whether there were any misunderstandings or gaps in communication that affected your care, and whether language barriers or other factors caused any issues. Reflect on whether the medical staff appeared well-coordinated and if the exchange of information between them contributed to your feeling of safety and being well cared for.

<u>The physicians were well informed about my history and current medical condition and treatment.</u>
Explanation: Consider whether they had easy access to your medical records, demonstrated familiarity with your condition, and were able to answer your questions comprehensively. Reflect on whether digital tools were effectively used to support information exchange, both within the hospital and with other facilities, and how well physicians communicated your treatment plan in a clear and understandable way.

<u>The nurses were well informed about my history and current medical condition and treatment.</u>
Explanation: Consider whether they appeared to have access to your medical records, demonstrated knowledge of your condition, and whether they were able to answer your questions about your care. Also, reflect on whether they used digital tools or monitoring devices effectively and how their communication and attention to detail influenced your confidence in their expertise and the quality of your care.

<u>After handover (shift change, transfer), staff knew all relevant information necessary for my care.</u>

Explanation: Consider whether you felt the handover was smooth, with no need to re-explain your concerns, and if the staff immediately took appropriate actions. Reflect on whether any important details were overlooked and how effectively the staff responded to your ongoing needs during these transitions, particularly when different staff members were involved in your care.

<u>Physicians and nurses worked together as a well-rehearsed team.</u>

Explanation: Consider whether communication was respectful and fluid, whether there was a division of responsibilities between the professions, and whether you felt the team worked together effectively and harmoniously despite differences in hierarchy.

<u>The different services (ward, x-ray, physiotherapy, etc.) are well coordinated.</u>

Explanation: Consider whether there was a smooth flow of care, whether services were well-timed, and if delays or miscommunications occurred between departments that affected your treatment. Reflect on whether the different departments collaborated efficiently to ensure a seamless patient experience.

<u>I always knew who was responsible for my treatment and care.</u>

Explanation: Consider whether it was clear to you which doctors and nurses were in charge, especially during shift changes or when new staff members joined. Reflect on whether this clarity helped you feel more secure or, conversely, if frequent changes in staff caused confusion or uncertainty about who was responsible for your care.

<u>Staff freely spoke up whenever they had the impression that something was amiss.</u>

Explanation: Consider whether the nurses and doctors communicated transparently when something seemed amiss, if they acknowledged and corrected mistakes, and if they responded quickly to any issues you raised. Reflect on whether you felt the staff was honest and direct with you, as this can impact your overall trust in the care you received.

<u>There was always enough qualified staff available.</u>

Explanation: Consider both the number of staff and their level of experience and competence. Reflect on whether you felt well-supported by the staff, especially in challenging situations, and whether there was adequate supervision for less experienced team members. Additionally, note if you felt there were any concerns regarding staffing levels or if the combination of experienced and less experienced staff affected the quality of your care.
